# Severe mitochondrial encephalomyopathy caused by *de novo* variants in *OPA1* gene

**DOI:** 10.3389/fgene.2024.1437959

**Published:** 2024-08-20

**Authors:** Michela Di Nottia, Teresa Rizza, Enrico Baruffini, Claudia Nesti, Alessandra Torraco, Daria Diodato, Diego Martinelli, Flavio Dal Canto, Alexandru Ionut Gilea, Martina Zoccola, Barbara Siri, Carlo Dionisi-Vici, Enrico Bertini, Filippo Maria Santorelli, Paola Goffrini, Rosalba Carrozzo

**Affiliations:** ^1^ Unit of Cell Biology and Diagnosis of Mitochondrial Disorders, Laboratory of Medical Genetics, Bambino Gesù Children’s Hospital, IRCCS, Rome, Italy; ^2^ Neuromuscular Disorders Research Unit, Bambino Gesù Children’s Hospital, IRCCS, Rome, Italy; ^3^ Department of Chemistry, Life Sciences and Environmental Sustainability, University of Parma, Parma, Italy; ^4^ Molecular Medicine, IRCCS Stella Maris Foundation, Pisa, Italy; ^5^ Division of Metabolic Diseases and Hepatology, Bambino Gesù Children’s Hospital IRCCS, Rome, Italy

**Keywords:** *OPA1*, mitochondrial diseases, encephalomyopathy, mitochondrial dynamics, optic atrophy

## Abstract

**Background:**

Mitochondria adjust their shape in response to the different energetic and metabolic requirements of the cell, through extremely dynamic fusion and fission events. Several highly conserved dynamin-like GTPases are involved in these processes and, among those, the OPA1 protein is a key player in the fusion of inner mitochondrial membranes. Hundreds of monoallelic or biallelic pathogenic gene variants have been described in *OPA1*, all associated with a plethora of clinical phenotypes without a straightforward genotype-phenotype correlation.

**Methods:**

Here we report two patients harboring novel de novo variants in *OPA1*. DNA of two patients was analyzed using NGS technology and the pathogenicity has been evaluated through biochemical and morphological studies in patient’s derived fibroblasts and in yeast model.

**Results:**

The two patients here reported manifest with neurological signs resembling Leigh syndrome, thus further expanding the clinical spectrum associated with variants in *OPA1*. In cultured skin fibroblasts we observed a reduced amount of mitochondrial DNA (mtDNA) and altered mitochondrial network characterized by more fragmented mitochondria. Modeling in yeast allowed to define the deleterious mechanism and the pathogenicity of the identified gene mutations.

**Conclusion:**

We have described two novel-single *OPA1* mutations in two patients characterized by early-onset neurological signs, never documented, thus expanding the clinical spectrum of this complex syndrome. Moreover, both yeast model and patients derived fibroblasts showed mitochondrial defects, including decreased mtDNA maintenance, correlating with patients’ clinical phenotypes.

## 1 Introduction

The ability of mitochondria to meet the energy needs of the cell depends on their dynamism, determined by regulated cycles of fusion and fission. The fusion process relies on the action of dynamin-related GTPases, which are responsible for the tethering of both inner and outer mitochondrial membranes. The fusion of inner mitochondrial membranes is controlled by OPA1, a dynamin-like GTPase which displays a mitochondrial-targeting sequence, a predicted transmembrane domain, followed by the three highly conserved regions: the GTPase, the middle and the GTPase effector (Gao and Hu, 2021). Besides the remodeling of the mitochondrial network, OPA1 protein is also involved in oxidative phosphorylation, mitochondrial genome maintenance, as well as in the regulation of cytochrome c release during apoptosis (Lee and Yoon, 2018). Monoallelic mutations in *OPA1*, have been recognized as the major cause of the autosomal dominant optic atrophy (DOA) (DOA, MIM#165500), in most cases manifesting with an isolated optic atrophy. About 20%–30% of DOA patients also develop additional clinical manifestations including severe sensorineural deafness, cerebellar ataxia, myopathy, peripheral neuropathy, spastic paraplegia, multiple sclerosis-like syndrome or clinical features of parkinsonism and dementia (so termed “DOA-plus”, OMIM #125250) (Verny et al., 2008; Yu-Wai-Man et al., 2010; Carelli et al., 2015). Biallelic *OPA1* inheritance has been associated with the severe early-onset Behr syndrome, Leigh-like and MELAS-like stroke syndromes (Chao de la Barca et al., 2016; Nasca et al., 2017; Rubegni et al., 2017; Zerem et al., 2019). Leigh syndrome (LS, # 256000), a subacute necrotizing neurometabolic disorder, is estimated to be the most common pediatric manifestation of mitochondrial disease. It is characterized by infantile onset of neurologic features such as hypotonia, spasticity, movement disorders and peripheral neuropathy. Decompensation (often with high lactate levels in blood and/or cerebrospinal fluid) during an intercurrent illness is usually associated with psychomotor retardation or regression. Extraneurologic manifestations may include heart, liver, large intestine and renal involvement. Both biochemical and genetic heterogeneity is documented in LS.

Since the last update in August 2023, the gene specific mutation database (https://www.lovd.nl/OPA1) counts 681 unique public variants associated with human diseases. Although a clearcut genotype-phenotype correlation is still missing and limited by the uneven use of functional investigations, a more severe clinical phenotype seems to be associated with monoallelic missense mutations in the GTPase and dynamin domains, whereas severe encephalopathy and early death are more common in the cases of *OPA1* bi-allelic inheritance (Xu et al., 2021).

Here we report two patients harboring novel *de novo* heterozygous variants in *OPA1*, manifesting with severe Leigh-like neurological signs.

## 2 Methods

### 2.1 Standard protocol approvals, registrations, and patient consent

The study was approved by the Ethical Committees of the Bambino Gesù Children’s Hospital, Rome (Italy), and the Fondazione Stella Maris, Pisa (Italy), in agreement with the Declaration of Helsinki. Informed consent for molecular genetic analysis was obtained from the patient’s parents.

### 2.2 Genetic studies

Total DNA from patient and parents was isolated using the QIAamp DNA Mini Kit (Qiagen, Valencia, CA, United States).

For Patient 1 the DNA was analyzed using the TruSightOneExpanded panel (clinical exome, Illumina, San Diego, CA, United States), comprehensive of greater than 6.700 clinically relevant genes, whereas for Patient 2 genetic analysis used a customized multigene next-generation sequencing (NGS) panel for mitochondrial diseases (Tolomeo et al., 2021) (see Supplementary Material). The libraries underwent high‐throughput sequencing on a NextSeq 500 System (Illumina, San Diego, CA, United States). For both patients, prioritization of the variants was achieved by selecting variants having allele frequency < 0.01 according to public database (Exome Variant Server, http://evs.gs.washington.edu/EVS; gnomAD, http://gnomad-old.broadinstitute.org; ExAC, http://exac.broadinstitute.org) or in-house database, and considering quality scores (e.g., coverage > 20), amino acid impact (High or Moderate according to *in silico* prediction tools such as PolyPhen2, SIFT, FATHMM, REVEL, EVE), and possible clinical significance. Standard Sanger sequencing was performed to confirm the presence of the identified variants in the proband and to assess segregation in the families.

### 2.3 Quantitative real-time polymerase chain reaction (qPCR)

Total RNA was isolated from control and patients’ fibroblasts with the Total RNA Purification Plus Kit (Norgen Biotek Corp, Canada). One µg of total RNA was reversely transcribed using LunaScript RT Super Mix Kit (New England Bio Labs) and qPCR performed using the TaqMan Universal PCR Master Mix (Applied Biosystems, Paisley, United Kingdom) and the TaqMan probe for OPA1 (Hs01047013_m1), while the relative abundance of OPA1 transcript was normalized to the expression level of GAPDH (Hs02786624_g1). Data were analyzed using the 2^−ΔΔCT^ method and reported as fold change relative to controls.

### 2.4 Biochemical studies

Human primary fibroblasts were obtained from skin biopsy and grown in Dulbecco’s modified Eagle medium (DMEM, high glucose formulation), supplemented with 10% fetal bovine serum, 50 μg/mL uridine and 110 mg/L sodium pyruvate.

Protein expression was studied by western blot. Cell extracts were separated by electrophoresis, using pre-cast 4%–12% sodium dodecyl sulfate–polyacrylamide gel (Thermo Fisher) and transferred to polyvinylidene difluoride membranes (PVDF) (Thermo Fisher). PVDF membranes were incubated with the following antibodies: anti-OPA1 (1:1,000, BD Biosciences), anti-complex I NDUFS1 (1:1,000, Santa Cruz), anti-complex II SDHA, anti-complex III -UQCRC2, anti-complex IV MT-COX2 and anti-complex V ATP5PD (1:1,000, Abcam), anti-TOMM20 (1:1,000, SantaCruz) and anti-rabbit or anti-mouse horseradish peroxidase–coupled secondary antibody (1:30,000, Jackson Immuno-Research). Bands detection was performed using Clarity Western ECL Substrate (Bio-Rad). Densitometry analysis was performed using Image Lab software (Bio-Rad).

For evaluation of mitochondrial ATP content, 5 × 10^3^ fibroblasts were seeded in a 96-well plate. The following day the cells have been fed either with regular or with glucose-free DMEM medium supplemented with 5 mM galactose for 24 and 48 h. The content of cellular ATP was assayed using the ATPLITE 1 STEP kit (PerkinElmer) according to the procedure recommended by the manufacturer. Luminescence was measured using the EnSpire Multimode Plate Readers (PerkinElmer, United States). All values were normalized to cell number as measured by Crystal Violet (Sigma-Aldrich).

### 2.5 Mitochondrial morphology

Healthy control and patients’ fibroblasts were seeded on 35 mm glass-bottom tissue culture plates. Cells were grown either in regular or in a restrictive glucose-free DMEM medium supplemented with 10% dialyzed FBS and 5 mM galactose for 24, 48 and 72 h. For immunofluorescence staining, cells were fixed and permeabilized using methanol:acetone (2:1, v/v) for 10 min at room temperature. To minimize background signals, non-specific antigens were blocked with a solution containing 5% bovine serum albumin in phosphate‐buffer. The primary polyclonal rabbit TOMM20 antibody (Santa Cruz Biotechnology) was incubated in 4% bovine serum albumin in PBS overnight at 4°C. Cells were washed three times and incubated with secondary antibody (Alexa-568 fluorophore, Molecular Probes) for 1 h at room temperature. For nuclei staining cells were incubated with Hoechst 33258 (Sigma-Aldrich) for 5 min at room temperature. Images were collected and analyzed on a fluorescence‐inverted microscope (Leica DMi8, Leica Microsystems., Germany).

### 2.6 Yeast studies

The yeast strains used in this work are reported in Supplementary Table S1. Media, growth conditions and construction of the mutant alleles were reported in the Supplementary Materials and Methods. Yeast functional studies, that is oxidative growth assay, oxygen consumption rate (OCR), mitochondrial DNA stability, and analysis of mutation inheritance, were performed as previously reported and detailed in Supplementary Material (Del Dotto et al., 2018; Cappuccio et al., 2021; Ceccatelli Berti et al., 2021).

## 3 Results

### 3.1 Clinical investigation

**FIGURE 1:**
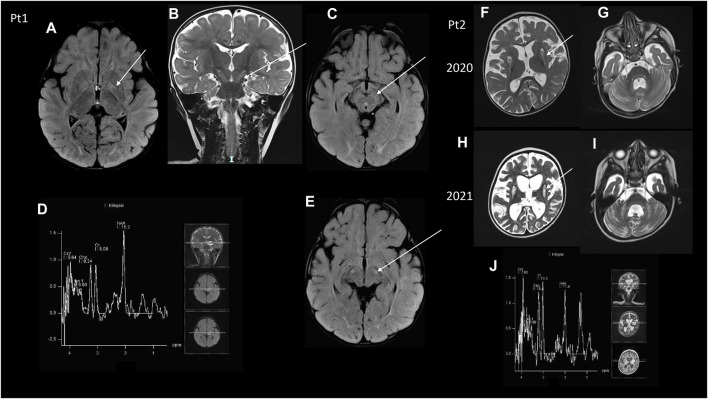
MRI **(A–C,E)** Brain MRI of Pt1. **(A)** Axial image showing FLAIR hyperintensities at the level of the pallidus bilaterally (arrow) and posterior white matter. **(B)** Coronal T2 image showing hyperintensities of the cortical spinal tracts (arrow), that was also evident at the peduncles of the midbrain in two axial FLAIR images **(C,E)**. **(D)** MRS did not show any clear abnormal lactate peak. **(F–I)** Brain MRI of Pt2, two serial MR images. **(F,G)** The first MRI was taken in 2020 and showed diffuse atrophy of the brain and of the cerebellum together with hyperintensity at the caudate nucleus bilaterally, cerebellar peduncles, bilateral cerebellar deep white matter at T2 weighted images. **(H,I)** Serial control brain MRI images showed a worsening of the brain cortical atrophy, compared to the previous MRI examination at T2 weighted images, with the absence of a clear lactate peak at MRS **(J)**.

Patient 1 is a baby boy born at 39 weeks to non-consanguineous parents by cesarean section after a normal pregnancy. Apgar scores were 8 and 9, at minutes 1 and 5, respectively. Perinatal period was normal. At the age of 3 months, a convergent strabismus appeared in the right eye, then nystagmus appeared in both eyes; he then performed an ophthalmologic examination that revealed bilateral pallor of the optic discs. Psychomotor delay was seen since the age of 8 months. At 11 months of age, once hospitalized, neurologic examination revealed horizontal nystagmus, convergent strabismus, hypotonia, psychomotor delay; the patient was able to hold his head, but he did not maintain the sitting position. Laboratory analyses revealed increased plasma (5.95 mmol/L, normal value 0.9–1.7) and CSF lactate (3.96 mmol/L, normal value 1,1–2,8); urinary organic acids, and plasma amino acids were normal; FGF21 showed a 4-fold increase (830 pg/mL, normal value 0.0–200), while GDF15 was in the normal range. Visual evoked potentials (VEP) showed abnormal bilateral complex N1/P2 definition and brainstem evoked potential (BAEPs) showed poor definition of pontine response, whereas electroretinogram (ERG) and electroneurography were normal. Brain MRI (Figures 1A–C) disclosed hyperintense T2/FLAIR signal in the pallidus bilaterally, corticospinal tracts, brainstem, and subcortical white matter in the occipital region. A muscle biopsy showed normal histochemical reactions for COX and SDH, useful tools in the context of mitochondrial diseases. During the follow-up the child gained the ability to maintain the sitting position, to roll and to reach the objects even though with some uncertainty. On the other hand, he developed progressive feeding difficulties and dysphagia, and he underwent gastrostomy at 18 months of age. He then experienced recurrent seizures and was put on therapy with Levetiracetam 800 mg/BID. The boy had recurrent episodes of epileptic seizures/dystonias appearing mostly at night associated with tachycardias, but with no metabolic decompensation. During the following years the neurological condition has slowly worsened with global motor regression, hyperkinetic and chaotic movements in upper limbs, and nystagmus. The last neurological examination was performed at 4 years of age, and the child was intermittently alert and reactive with smiling, and had a with spastic-dystonic tetraparesis with nystagmus in all gaze directions.

Patient 2 is a baby girl born from non-consanguineous parents after a pregnancy complicated by pre-eclampsia and IUGR. At birth marked hypotonia was noted, but she had no respiratory problems. During the follow up she had marked psychomotor delay and never achieved a sitting position, but she had been able to roll on one side. At 15 months of age, she presented with respiratory distress, characterized by dyspnea and hypercapnic acidosisand metabolic derangement, and she was admitted to the pediatric ICU where she had a gastrostomy and non-invasive ventilation was started. Metabolic analyses showed slightly increased plasma lactate (2.54 mmol/L), normal urinary organic acids, plasma amino acids and acylcarnitine profile. FGF21 was slightly elevated (305 pg/mL, normal value 0–200). GDF15 was in the normal range. Cardiologic evaluation with ECG and echocardiography was normal. A brain MRI disclosed T2/FLAIR hyperintense signal of subcortical white matter and bilateral striatal necrosis with brainstem lesion and Leigh syndrome was hypothesized (Figures 1D,E). She also performed electroneurography showing signs of axonal polyneuropathy; VEP showed increased latency in OO, ERG was normal, and BAEPs were absent bilaterally. At that time neurologic examination revealed absent visual fixation and tracking, marked hypotonia with absence of anti-gravity movements in the lower limb; the child was not able to hold her head or to maintain the sitting position, but was only able to reach her mouth with her arms when in supine position. Antioxidant treatment with CoQ10 400 mg/BID, Riboflavin 10 mg/kg/day and Idebenone 10 mg/kg/day was started. At the age of 2 years, the patient presented myoclonic status epilepticus. EEG disclosed a periodic pattern of slow waves with burst suppression phases; treatment with phenobarbital and levetiracetam was started with resolution of the acute phase. Then phenobarbital was gradually reduced and stopped, maintaining Levetiracetam treatment. The motor function worsened in the upper limb with absence of anti-gravity movements. At age 3 a novel brain MRI revealed T2 hyperintensity at the caudate nucleus bilaterally and overall worsening of her encephalopathy (Figures 1F–I). The last neurological examination, at the age of 3 years and a half, showed tetraparesis with spasticity in the lower limb.

### 3.2 Molecular genetics

**FIGURE 2:**
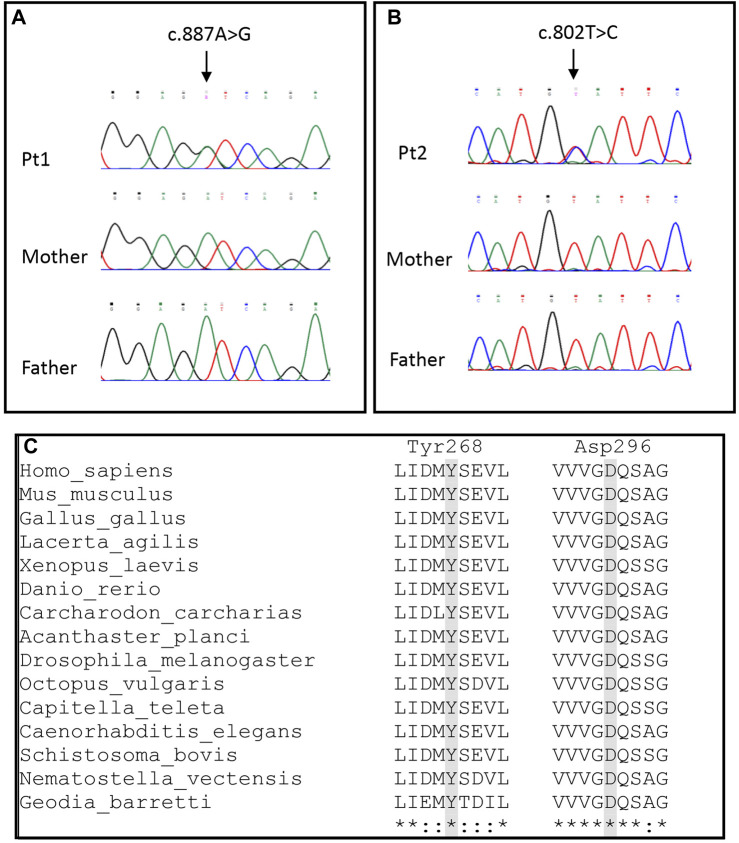
Molecular findings. **(A,B)** Electropherograms of Pt1 and Pt2 showing the c.887A > G and c.802T > C variants, respectively. **(C)** Alignments of OPA1 sequences of different metazoan phyla and classes. *Homo sapiens* and *Mus musculus* (Mammalia), *Gallus gallus* (Aves), *Lacerta agilis* (Reptilia), *Xenopus laevis* (Amphibia), *Danio rerio* (Osteichthyes), *Carcharodon carcharias* (Chondrichthyes), *Acanthaster planci* (Echinodermata), *Drosophila melanogaster* (Arthropoda), *Octopus vulgaris* (Mollusca), *Capitella teleta* (Annelida), *Caenorhabditis elegans* (Nematoda), *Schistosoma bovis* (Platyhelminthes), *Nematostella vectensis* (Cnidaria) and *Geodis barretti* (Porifera).

Patient 1 harbored a *de novo* heterozygous variant, c.887A > G (p.Asp296Gly) (Figure 2A) in *OPA1* (NM_ 015560.3) never reported before, although another substitution (c.888T > A; p.Asp296Glu, annotated according to NM_130837.3 as c.1053T > A; p.Asp351Glu) in the same amino acidic position has been reported in a family with a variable age of onset of DOA plus phenotype (Ahmad et al., 2015). No other rare or unique variants in genes associated with the HPO terms were found.

Patient 2 had a *de novo* heterozygous variant, c.802T > C (p.Tyr268His) (Figure 2B) in *OPA1* (NM_015560.3). The residue Tyr268 has been reported to be affected by a different substitution (p.Tyr268Cys) in a patient with familiar optic atrophy (Chen et al., 2014). Moreover, Patient 2 harbored the rare synonymous heterozygous variant c.381G > A (p.Pro127Pro) (MAF 3.2 × 10^−5^; no homozygotes reported in gnomAD database), inherited from the healthy father. The substitution is located in the middle of exon 3 and it is not predicted to produce alteration of splicing.

Both p.Asp296Gly and p.Tyr268His occur in the highly conserved GTPase region of *OPA1*, were not reported in public databases (gnomAD, ExAc, 1000Genomes), had a high CADD score, and can be classified as “pathogenic” according to the standard ACMG nomenclature (criteria PS2 + PM1 + PM2 + PP3 + PM5). Both amino acids are conserved in all deposited metazoan sequences, from Porifera to Mammalia (Figure 2C). Conversely, the p.Pro127Pro in Patient 2 had a low CADD score (i.e., Tolomeo et al., 2021) and it was classified as likely benign according to the ACMG guidelines.

### 3.3 Functional analysis on fibroblasts

**FIGURE 3:**
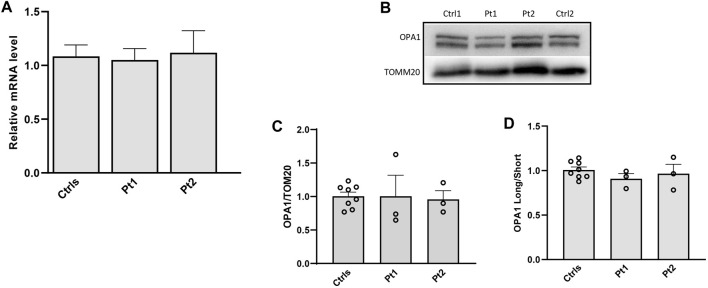
Transcript level and protein amount of OPA1 in patients’ fibroblasts. **(A)** Expression level of *OPA1* transcript; **(B)** Western blot of OPA1, a representative experiment out of three is shown; TOMM20 was used as loading control. **(C,D)** Densitometric analysis of OPA1/TOMM20 and OPA1 long/short forms ratio. Data from patients’ fibroblasts (Pt1, Pt2) were compared to the mean value of controls (=1) and are expressed as mean ± SEM (n = 3).

**FIGURE 4:**
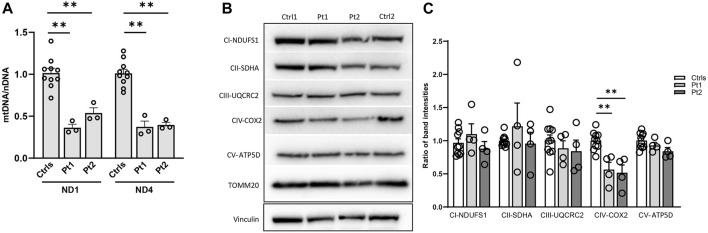
Impact of OPA1 defect on mtDNA amount and mitochondrial complex (MC) subunits level in patients’ fibroblasts. **(A)** Quantification of mtDNA amount in fibroblasts from patients (Pt1, Pt2) and controls (Ctrls). The amount of mtDNA was normalized to nuclear DNA and compared to the mean value of controls (=1). Two different probes for mtDNA were used (*ND1*, *ND4*). Data are represented as mean ± SEM of Ctrls (n = 10) and patients (n = 3) **:*p* < 0.005 in Mann-Whitney test. **(B)** Western blot of mitochondrial complexes subunits, a representative experiment out of three is shown; TOMM20 was used as loading control. **(C)** Densitometric analysis of mitochondrial complexes subunits/TOMM20. Data from patients’ fibroblasts (Pt1, Pt2) were compared to the mean value of controls (=1) and are expressed as mean ± SEM (n = 4). **:*p* < 0.005 in Mann-Whitney test.

**FIGURE 5:**
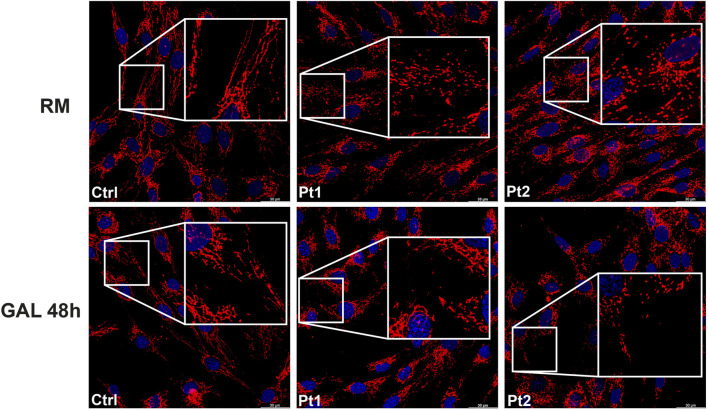
Mitochondrial morphology in patients’ fibroblasts. Representative images of mitochondrial morphology in fibroblasts from control (Ctrl) and patients (Pt1, Pt2), labeled with TOMM20 antibody. Cells were grown either in regular medium (RM) (upper panel) or galactose medium (GAL) for 48 h (lower panel). Scale bar: 30 μm.

**FIGURE 6:**
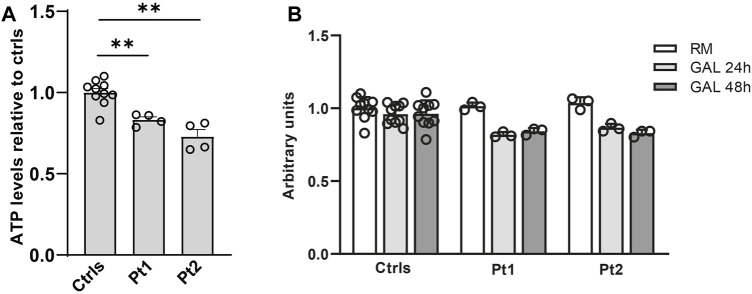
Energetic status evaluation in patients’ fibroblasts. **(A)** Total cellular ATP content at basal conditions. The level of ATP content was normalized to cells number measured by Crystal Violet and compared to the mean value of controls (=1). Data are represented as mean ± SEM of Ctrls (n = 10) and patients (n = 4). **:*p* < 0.005 in Mann-Whitney test. **(B)** Cellular ATP content in controls and patients’ fibroblast grown either in regular medium (RM) or in galactose medium (GAL) for 24 and 48 h. The level of ATP content was normalized to cells number measured by Crystal Violet and treated fibroblasts (GAL 24 and 48 h) were compared to the mean value of untreated fibroblasts (RM) (=1); the results were not significant using the Wilcoxon test.

In patients’ and controls’ cultured skin fibroblasts, we observed similar transcript levels and amount of OPA1 protein (Figures 3A–C). Quantification of the relative amount of OPA1 long and short forms revealed unchanged long/short forms ratio for both patients (Figure 3D). The amount of mtDNA was about 50% in both patients respect to control fibroblasts (Figure 4A). The measurement of mitochondrial complex subunits level by western blot, did not show differences compared to controls, except for the mitochondrially-encoded COX2 subunit of complex IV, probably due to the depletion of mtDNA we observed in patients’ fibroblasts (Figures 4B,C). Given the central involvement of OPA1 in the mitochondrial fusion process, we analyzed the mitochondrial morphology in fibroblasts. Patients’ cells, cultured in standard glucose-medium, showed an altered mitochondrial network characterized by more fragmented mitochondria compared to control fibroblasts (Figure 5, upper panel). A similar mitochondrial morphology is evident when cells are grown in galactose-supplemented medium (Figure 5, lower panel), indicating a lack of additional effect of galactose. In addition, we analyzed the energetic status of the cells and we found in patients’ fibroblasts decreased levels of ATP at basal conditions (Figure 6A). Moreover, the replacement of glucose with galactose for 24 and 48 h, as carbohydrate source, produced a mild reduction, though not significant, in patients respect to controls cells (Figure 6B).

### 3.4 Functional studies in yeast

**FIGURE 7:**
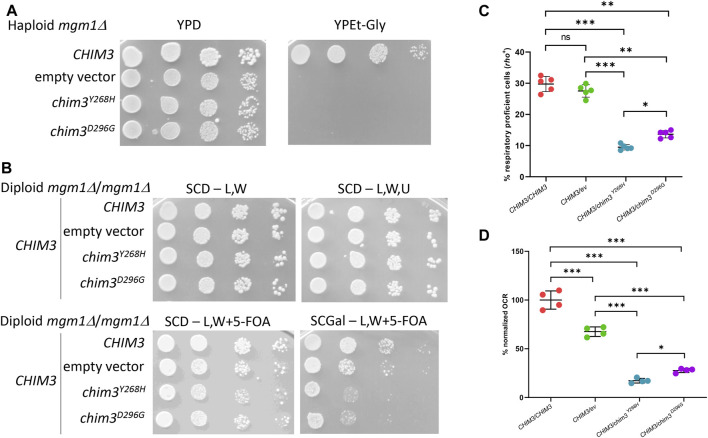
Modeling of the *OPA1* mutations in yeast. **(A)** Spot assay of the haploid *mgm1Δ* strain transformed with: *CHIM3* allele cloned in pFL39TEToff, the empty vector or the *chim3* mutant alleles in the same vector. The growth was analyzed on YP medium supplemented with either 2% glucose (YPD) as a control or 2% ethanol and 2% glycerol (YPEt-Gly) and plates were incubated at 28°C for 2–4 days. **(B)** Spot assay of the diploid *mgm1Δ*/*mgm1Δ* strain transformed with plasmid pFL38MGM1, *CHIM3* allele cloned in the vector pFL36TEToff, and *CHIM3* allele cloned in pFL39TEToff, the empty vector or the *chim3* mutant alleles in the same vector. Plates were incubated at 28°C for 3–5 days. SCD-L,W medium is SC medium supplemented with 2% glucose without leucine and tryptophan, SCD-L,W,U the same medium without uracil. Similar growth among the different clones on SCD-L,W and SCD-L,W,U plates confirmed that the number of plated cells was similar before and after the loss of pFL38MGM1. SCD-L,W + 5-FOA medium is the same medium as in the first panel but with the supplementation of 5-fluoroorotic acid. Similar growth on this medium confirmed that, after the loss of pFL38MGM1, the clones showed similar fermentative growth. SCGal-L,W + 5-FOA medium contained galactose instead of glucose. Heteroallelic strains showed a decreased growth compared to both homoallelic and hemiallelic strains, indicating that the respiratory growth is decreased. **(C)** Frequency of cells that are respiratory proficient, or *rho* ^*+*^, after growth on medium supplemented with the fermentable carbon source glucose. The strains are the same as in panel **(B)**. Five independent clones for each strain were analyzed. **(D)** OCR activity of the same strains as in panel **(B)**. The oxygen consumption of each clone was at first normalized for the dry weigh of the cells and then for the absolute OCR of the *CHIM3*/*CHIM3* homoallelic strain. At least four independent clones for each strain were analyzed. *:*p* < 0.05; **:*p* < 0.01; ***:*p* < 0.001 in one-way ANOVA followed by a Bonferroni’s *post hoc* test.

To assess the pathogenic role of the identified *OPA1* variants, we performed complementation studies in *Saccharomyces cerevisiae* strains lacking the orthologous gene *MGM1*, hereafter referred to as *mgm1Δ*. Lack of Mgm1 activity is associated with mitochondrial fusion defects, round mitochondria with absent cristae and to the loss of nucleoids and of mtDNA, with a consequent respiratory deficient phenotype (Wong et al., 2000; Amutha et al., 2004; Meeusen et al., 2006; Merz and Westermann, 2009; Zick et al., 2009; Schäfer et al., 2010). The mutations p.Tyr268His and p.Asp296Gly were introduced in a human *OPA1*-yeast *MGM1* chimeric gene, called *CHIM3* (Nolli et al., 2015), obtaining *chim3* ^*Y268H*^ and *chim3* ^*D296G*^ mutant alleles. Contrary to the *CHIM3* wild type allele, when the mutant alleles were introduced in a haploid *mgm1Δ* strain, they were not able to complement the loss *MGM1*, since no oxidative growth was observed (Figure 7A). To evaluate whether the respiratory deficient phenotype was due to the lack of whole mtDNA molecules, which means that the cells are *petites*, we crossed the two mutant strains with a tester strain of the opposite mating type deleted in *MIP1*, encoding for the mitochondrial DNA polymerase, and thus depleted in mtDNA (*rho* ^*0*^) and respiratory deficient. It is then expected that, after crossing, the tester strain complemented the lack of *MGM1*, whereas the tested strain complemented the lack of *MIP1*. The diploid strains obtained will be thus respiratory proficient if the tested strains are *rho* ^*+*^, that is, if they contained whole molecules of mtDNA, or respiratory deficient if they are *petites*. The diploid strains transformed with the empty vector or with the *chim3* ^*Y268H*^ and *chim3* ^*D296G*^ mutant alleles were respiratory deficient, indicating that both Chim3 protein variants are unable to maintain mtDNA (data not shown).

To evaluate whether the mutations are recessive or dominant, and the kind of dominance, diploid strains were obtained transforming a *mgm1Δ*/*mgm1Δ* strain, containing pFL38MGM1, with both a plasmid harboring the *CHIM3* wild type allele and a plasmid harboring the *CHIM3* wild type allele, the *chim3* mutant alleles or the empty plasmid. It has been previously demonstrated that the presence of a single copy of *CHIM3* in a diploid strain (hemiallelic strain) caused haploinsufficiency, showing decreased oxidative growth compared to the strain harboring two copies of *CHIM3* (homoallelic strain) (Nolli et al., 2015). It is expected that if the phenotype of strains harboring a *CHIM3* wild type allele and a *chim3* mutant allele (heteroallelic strains) is similar to that of the hemiallelic strain, the allele is null, resulting in humans in a dominance by haploinsufficiency; if the phenotype is worse than the hemiallelic strain, the mutation is dominant by gain-of-function or, more likely, is negative dominant (Ceccatelli Berti et al., 2021). The loss of the plasmid pFL38MGM1 was induced as reported in Material and Methods. SCD medium without leucine and tryptophan allowed the growth of cells that have lost or not pFL38MGM1 plasmid, but only if they have maintained both *CHIM3* wild-type and *chim3* mutant alleles. The growth on SCD medium without leucine, tryptophan and uracil allowed the growth only of cells that have maintained each of the three plasmids. In both media, the growth was similar for all strains, suggesting that a similar number of spotted cells has lost pFL38MGM1.

When 5-fluoroorotic acid (5-FOA) was present in the media, only cells that have lost pFL38MGM1 can grow, since the product of the marker gene harbored by this vector, *URA3*, converts 5-FOA in a toxic drug which inhibits yeast growth. When a fermentable carbon source such as glucose was present in the 5-FOA medium, the growth was similar for all strains. However, on medium containing galactose, which in SC medium with 5-FOA is mainly oxidized in order to obtain ATP, the growth of the heteroallelic strains was decreased compared not only to the growth of the homoallelic strain, but also to that of the hemiallelic strain (Figure 7B). This result indicates that the mutations are dominant, and the dominance is not due to haploinsufficiency, but likely to the interference of the Chim3 protein variant with the Chim3 wild type protein. This kind of dominance was confirmed by the measurement of the frequency of the colonies that are respiratory proficient and thus *rho* ^*+*^: in fact, in the heteroallelic strains the frequency of *rho* ^*+*^ colonies was lower compared to that of the hemiallelic strain (Figure 7C). Finally, the oxygen consumption rate (OCR) of the heteroallelic strains was also decreased compared to the hemiallelic strain (Figure 7D). Interestingly, the p.Tyr268His variant is associated with a more detrimental phenotype compared to p.Asp296Gly variant, suggesting that the former mutation is more deleterious than the latter. Altogether, the modeling in yeast confirmed that the mutations found in patients are pathogenic and dominant, most likely due to a negative dominance.

## 4 Discussion

To date over 600 pathogenic variants in the *OPA1* gene have been described (Le Roux et al., 2019). Although the main clinical manifestation associated with monoallelic *OPA1* defect is optic atrophy, about 20%–30% of patients develop additional extraocular manifestations including a plethora of neurological features. These unusual signs typically are disclosed during young adulthood after optic neuropathy (Verny et al., 2008; Yu-Wai-Man et al., 2010; Carelli et al., 2015). Biallelic variants usually associate with earlier and more severe neurological manifestations in children (Othman et al., 2022).

In this report we described two patients with two novel, heterozygous mutations in *OPA1*, classified as “pathogenic” according to the standard ACMG nomenclature. The main point of interest in the present report is that both cases had a prominent neurological phenotype highly resembling clinical and neuroradiological features of Leigh-like though they harbored monoallelic variants in *OPA1*.

Both variants reported in this study affect the GTPase domain of the protein and missense mutations in this central domain are predicted to have both a higher risk of developing extraocular manifestations (Yu-Wai-Man et al., 2011; Yu-Wai-Man et al., 2016) and to lead to a more severe phenotype via a dominant-negative effect (Yu-Wai-Man et al., 2011; Ham et al., 2019). The missense variants we identified in this study are predicted to impair function of GTPase domain, whose integrity is required for inner mitochondrial membranes fusion (Olichon et al., 2006). Accordingly, we observed in patients’ fibroblasts more fragmented mitochondria and reduced ATP production compared to control fibroblasts, pointing out an energetic metabolism failure. Although with different traits, similar defects have been found in fibroblasts from different *OPA1* patients (Del Dotto et al., 2018). However, OPA1 was described to be also involved in both mtDNA integrity and copy number maintenance (Amati-Bonneau et al., 2008; Hudson et al., 2008; Elachouri et al., 2011), in fact mtDNA depletion has been documented in fibroblasts from patients with missense or compound heterozygous variants (Del Dotto and Carelli, 2021). However, mtDNA proliferation was once reported in leukocytes of patients with dominant optic atrophy and *OPA1* mutations; this result could be due, as suggested by the Authors, to the relatively short half-life of circulating leukocytes and their rapid turnover, which preclude the formation and subsequent clonal expansion of somatic mtDNA abnormalities (Sitarz et al., 2012). In keeping with the aforementioned data, we observed about 50% reduction of mtDNA in our patients respect to control fibroblasts. Complementary studies in yeast confirmed that the mutations detected in patients are pathogenic and most likely associated with a dominant negative mechanism, and this is in line with the unchanged level of OPA1 protein we observed in patients’ fibroblasts. Interestingly, both mutations, when modelled in heterozygosis with a wild type allele in yeast, decreased the respiratory activity by more than 70%, whereas a plethora of mutations associated with DOA and DOA-plus previously studied in the same model system had less aggressive phenotypes, indicating that the novel variants may be more deleterious. The occurrence of different manifestations of the *OPA1* mutations, including haploinsufficiency, negative dominance effect or hypomorphic variants, hampers a clear genotype/phenotype correlation but the availability of different disease models can largely overcome these difficulties (Del Dotto and Carelli, 2021).

An open question is why *OPA1*-related conditions can act through different pattern of transmission. It is noteworthy that carrier parents in autosomal recessive families are healthy in spite of a partially inactive gene product. Also, it seems that different protein domains appear to be critical for adequate functioning of OPA1 in fission mechanisms and for mediating its interactions with other proteins involved in mitochondrial dynamics. On the other hand, coexistence of autosomal recessive and dominant inheritance has been observed in families harboring mutations in other mitochondrial proteins (i.e., RRM2B, POLG, SPG7, etc.) suggesting that zygosity is no longer a barrier to defining a molecular diagnosis in mitochondrial disorders.

In conclusion, we have described two novel-single *OPA1* mutations in two patients characterized by early-onset neurological signs, never documented, thus expanding the clinical spectrum of this complex syndrome. In addition, functional studies on both patients derived fibroblasts and yeast model confirmed the pathogenic role of *OPA1* defect.

## Data Availability

The names of the repository/repositories and accession number(s) can be found in the article/Supplementary Material. The datasets presented in this study can be found in online repositories: Leiden Open Variation Database (LOVD): https://databases.lovd.nl/shared/individuals/00436738; https://databases.lovd.nl/shared/individuals/00436739.
